# Clinical Lectures on Diseases of the Eye

**Published:** 1865-12

**Authors:** E. L. Holmes

**Affiliations:** Chicago, Lecturer on Diseases of the Eye and Ear in Rush Medical College, and Surgeon to the Chicago Charitable Eye and Ear Infirmary


### CLINICAL LECTURES ON DISEASES OF THE EYE.

By E. L. HOLMES, M, D., of Chicago,

*Lecturer on Diseases of the Eye and Ear in Rush Medical College, and Surgeon to the Chicago Charitable Eye and Ear Infirmary.*

### THE ACCOMMODATION OF THE EYE FOR DISTINCT VISION AT DIFFERENT DISTANCES.

GENTLEMEN:

We enter upon the study of a very interesting series of subjects. I will ask your particular attention to this and the following Lectures, since there is comparatively little upon these subjects in your ordinary text-books. To the recent labors of Donders, Helmholz and Jaeger, we owe almost all the positive knowledge that now places this chapter of ophthalmology upon a strictly scientific basis.

The subject embraces the "accommodation" of the eye and its anomolies—Myopia, Presbyopia, Hypermetropia, Asthenopia, Astygmatism, and Paralysis and Spasm of the ciliary muscle.

I need scarcely remind you that the eye may be compared to the camera of the photographer, the cornea and other refracting media corresponding to the convex object glass, and the retina to the screen of ground glass, upon which the inverted images are produced. It is well to recall to mind the fact, that rays of light do not pass straight through convex lenses, but are rendered convergent as follows: Parallel rays converge at a distance behind the lens, equal to the length of the radius of its curvature. The more divergent, within certain limits, the rays are made to fall, the further behind the lens they will be brought to a focus. The rays from very distant objects, since they are so slightly divergent that they may be regarded for all practical purposes as parallel, form images on the screen placed at a distance behind the lens equal to the radius of its curvature. The nearer the objects (within certain limits,) are placed to the lens, the more divergent the rays from these become, and consequently the further their images are formed behind the lens. The photographer therefore slides his screen towards the lens for distant objects, or from it for nearer objects, and thus "accommodates" his camera for objects at different distances. You will recollect, again, that the greater the degree of convexity a lens posses-ses, the more it refracts the rays of light—or in other words, the shorter the distance behind it at which parallel rays are brought to a focus. If we suppose the screen and lens of the camera placed at a suitable fixed distance from each other, absolutely distinct images could be obtained of those objects only which were situated at one certain distance before the camera. Objects nearer or further than this distance would form images respectively behind or in front of the screen, and consequently imperfect images on the screen itself By substituting, however, for the lens in the camera, those of greater convexity, for nearer objects or of less convexity, for more distant objects, distinct images may be formed on the screen. Thus there are two ways by which the photographer may "accommodate" his camera, or in other words, change its focus—either by increasing or decreasing the distance between the lens and screen, or by substituting lenses of greater or less convexity.

I trust I shall be excused for dwelling thus long upon these simple illustrations. I think they will aid you in comprehending the subject of accommodation and the abnormal conditions of the eye, which disarrange this faculty.

The convex surfaces of the cornea and crystalline lens are the important elements in the refracting media of the eye which aid in forming images of external objects upon the retina.

It has been supposed by some physiologists that the degree of convexity of the lens as well as of the cornea was unchangeable; that the eye when looking upon distant objects was in a state of rest or relaxation; that in looking upon near objects the recti muscles so pressed upon the selerotic as to elongate the globe, thus increasing the distance between the retina and lens—as the photographer elongates his camera by removing the screen further from the lens.

This theory of accommodation is now almost wholly discarded by physiologists, for it has been shown that the recti muscles may be all paralyzed and yet the power of adjustment may remain intact.

Others have supposed that accommodation depended upon the motions of the pupil (iris,) since in looking at nearer objects the pupil contracts and the pupillary edge of the iris approaches slightly towards the cornea. This movement was supposed to draw the lens forward, thus increasing the distance between the lens and retina. It has been demonstrated however, that the iris may be torn from the eye and yet the power of accommodation be undisturbed.

It should, nevertheless, be borne in mind, that while the iris and recti muscles are in no way the essential agents of normal accommodation, they still perform their functions in true harmony with the organs of accommodation, the muscles rotating the eyes, and the iris regulating the amount of ligh which enters the eye, and also serving as a diaphragm. We shall soon have occasion to examine the influence of the recti muscles upon the accommodation of the eye in some forms of disease.

The normal adaptation of the eye for vision at different distances is effected by changes in the form of the crystalline lens. These changes have been demonstrated by eminent physiologists in a simple manner, which may be well illustrated by a familiar experiment. If two convex lenses, one of greater and the other of less convexity, be placed at a given distance from an object, the reflected image from the more convex will be smaller than that from the less convex lens. Hence the convex surface of the cornea gives a larger image of a luminous object than the convex surface of the crystalline lens, as can be readily seen when a lighted candle is held before a normal eye. If now the head of an individual and a suitable luminous object be fixed at a proper distance from each other, by an apparatus adapted for the purpose, the length of the image on the cornea, and that on the lens may be most accurately measured by a dilicate optical instrument of peculiar construction. It is found with this instrument that, while the cornea gives an image constantly of the same length whether the eye is accommodated for near or distant objects, the lens gives a shorter image when the eye is fixed upon near than when fixed upon distant objects. Hence it follows that the crystalline lens becomes *more* or *less* convex according as the eye is fixed upon near or distant objects.

From what has already been said, you perceive that the following changes take place in the eye when fixed upon near objects: the lens is more convex—the pupil contracts—the anterior portion of the lens and the pupillary edge of the iris approach slightly towards the cornea; the two internal recti muscles slightly contract to render the axes of the eye more convergent. The reverse of these changes takes place when the eyes are fixed upon distant objects.

The changes occurring in the form of the crystalline lens, as above described, are demonstrated beyond question. The precise manner, however, in which they are produced, is still somewhat difficult to comprehend. The ciliary muscle is now recognized by almost all authorities as the apparatus by which they are accomplished. The following explanation, although not entirely satisfactory, is, perhaps, the most rational that can be given:

An innate power of traction peculiar to the Zonula of Zinn or suspensary ligament of the lens, acting in all directions upon the periphery of the capsule of the lens, tends to increase its circumference, and consequently to flatten its convex surface, which is supposed to be the condition of rest of the organs of accommodation—the eye being thus adjusted for distant objects.

Whenever the lens is relieved from the traction of the zonula, it assumes, from its own inherent properties, a more convex form. It is a fact worthy of notice, that whenever the eonnection between the lens and zonula is ruptured, the lens at once becomes much more convex. After death, also, when the zonula has lost its power of traction, the lens immediately becomes more convex.

The contraction of the annular and radiating fibers of the ciliary muscle tends to shorten the width of the muscle, and to raise the ciliary processes towards the axis of the eye, thereby, as the zonula is attached to the processes, decreasing the periphery of the zonula. This removes the traction on the capsule of the lens, which at once, from its inherent tendency, assumes a more convex form, the eye thus being accommodated for near objects.

The plates which you have before you illustrate the changes in the form of the lens, and of the ciliary band in accommo dation for near and for distant objects.

